# Artificial Intelligence–Driven Computational Approaches in the Development of Anticancer Drugs

**DOI:** 10.3390/cancers16223884

**Published:** 2024-11-20

**Authors:** Pankaj Garg, Gargi Singhal, Prakash Kulkarni, David Horne, Ravi Salgia, Sharad S. Singhal

**Affiliations:** 1 Department of Chemistry, GLA University, Mathura 281406, Uttar Pradesh, India; 2 Department of Medical Sciences, S.N. Medical College, Agra 282002, Uttar Pradesh, India; 3 Department of Medical Oncology & Therapeutics Research, Beckman Research Institute of City of Hope, Comprehensive Cancer Center and National Medical Center, Duarte, CA 91010, USA; 4 Department of Molecular Medicine, Beckman Research Institute of City of Hope, Comprehensive Cancer Center and National Medical Center, Duarte, CA 91010, USA

**Keywords:** cancer drug development, artificial intelligence (AI), machine learning (ML), deep learning (DL), pharmacophore mapping, computer-aided drug design (CADD), molecular docking

## Abstract

The integration of AI has revolutionized cancer drug development, transforming the landscape of drug discovery through sophisticated computational techniques. AI-powered models and algorithms have enhanced computer-aided drug design (CADD), offering unprecedented precision in identifying potential anticancer compounds. Traditionally, cancer drug design has been a complex, resource-intensive process, but AI introduces new opportunities to accelerate discovery, reduce costs, and optimize efficiency. This manuscript delves into the transformative applications of AI-driven methodologies in predicting and developing anticancer drugs, critically evaluating their potential to reshape the future of cancer therapeutics while addressing their challenges and limitations.

## Simple Summary

This manuscript explores how artificial intelligence (AI) is revolutionizing the way to predict and develop drugs to fight cancer. Traditional methods of drug discovery can be lengthy and costly, but AI offers promising ways to accelerate and refine this process. By analyzing vast amounts of data, AI helps identify potential drug candidates, predict their effects, and tailor treatments to individual patients. The goal is to highlight how AI-driven approaches can bring faster, more effective cancer treatments to clinical practice. This report aims to benefit researchers, clinicians, and healthcare innovators by showcasing the potential of AI to advance anticancer drug development, ultimately leading to better outcomes for patients and a more efficient drug discovery process.

## 1. Introduction

Cancer is a complex group of diseases characterized by uncontrolled cell growth and proliferation. Globally, cancer remains one of the leading causes of death, with an estimated 20 million new cases and almost 9.7 million cancer-related deaths recorded in 2022 alone. The incidence and mortality rates vary significantly across regions, influenced by factors such as lifestyle, environment, and access to healthcare. In regions like North America and Europe, early screening and advanced treatments have contributed to relatively higher survival rates, although cancer burden continues to rise. In contrast, low- and middle-income countries, especially in Asia, Africa, and Latin America, experience high cancer mortality rates due to limited healthcare resources and late-stage diagnoses [1]. Developing effective drugs to treat cancer is challenging due to the intricate molecular interactions involved and the potential for drug resistance to develop [2]. Traditional experimental drug discovery methods are time-consuming and resource-intensive, making it imperative to incorporate computational approaches to streamline the process. The discovery of anticancer drugs involves a variety of computational methods that play crucial roles at different stages of the drug development process [3]. These methods help in identifying potential drug candidates, optimizing their properties, and understanding their interactions with cancer-related biomolecules (Table 1). The incorporation of computer-aided techniques for overseeing drug screening is becoming a pivotal element in the field of drug design [4]. This methodology empowers medicinal chemists to assess the interactions between a ligand and receptors, facilitating the design and refinement of lead compounds through computer simulations [5]. In drug design, the customary function of computer-aided design (CAD) is to scrutinize extensive compound libraries, grouping them into smaller clusters of anticipated active compounds through computational chemistry. This can significantly accelerate the anticancer drug design process and result in substantial time and cost savings [5].

**Figure 1:**
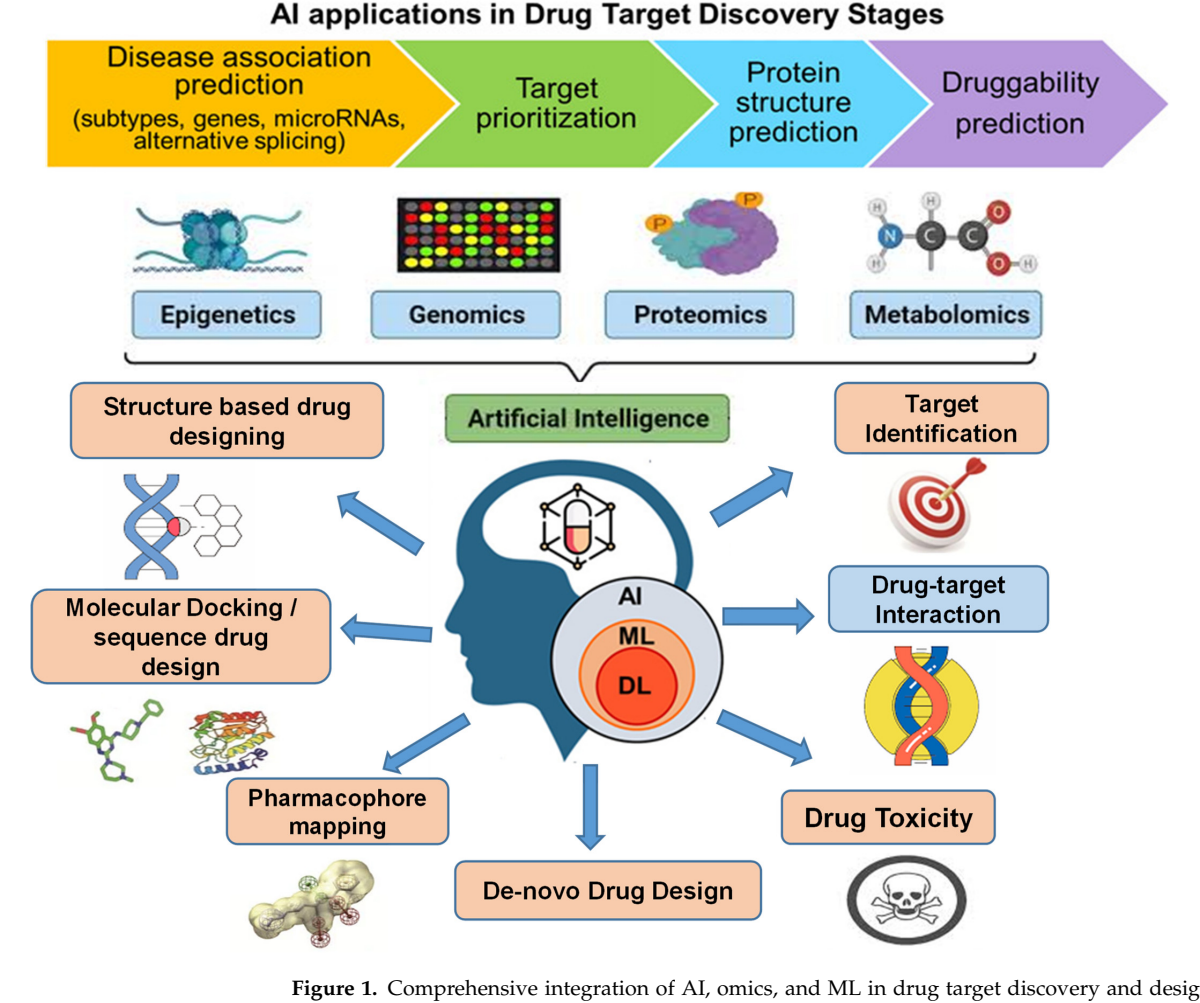
Comprehensive integration of AI, omics, and ML in drug target discovery and design. This figure highlights the transformative role of AI in drug target discovery, utilizing a multi-omics approach—epigenetics, genomics, proteomics, and metabolomics—to improve disease association prediction, target prioritization, protein structure prediction, and druggability prediction. AI, combined with ML and DL, accelerates key phases such as structure-based drug design, molecular docking, pharmacophore mapping, and de novo drug design. The figure emphasizes core stages like target identification, drug–target interaction, and drug toxicity assessment, with advanced AI techniques driving the development of more effective anticancer drugs. Figure creation source: Bio-Render software (https://biorender.com (accessed on 7 October 2024)).

Artificial intelligence (AI) involves replicating human intelligence in machines programmed to emulate human thought processes and actions [6]. A prevalent assumption regarding AI is its aim to create machines with a comparable ability to “understand” [7]. In contemporary cancer research, AI finds diverse applications, including the classification of abnormal cancer cells through image analysis, forecasting target protein structures, and anticipating interactions between drugs and proteins [8]. These investigations showcase the transformative potential of AI methods in reshaping processes related to anticancer drug design [9]. This report provides a comprehensive review of advancements in methods for designing anticancer drugs, specifically those leveraging AI [10]. Different applications of AI within the anticancer drug design processes have been illustrated in (Figure 1).

**Table 1:** Transformative applications of AI-driven software tools in drug discovery with representative drug examples.

AI-Driven Methodology	Application in Anticancer Drug Development	Key Advantages	Software Tools (Version/Website Access Details)	Drug/Compound Examples	Challenges and Limitations	References
Machine Learning (ML)	Predicting drug–target interactions, optimizing drug efficacy, identifying novel compounds	Reduces experimental costs and time, improves prediction accuracy	AutoDock Vina 1.2.x (Documentation), Chemprop (GitHub, https://github.com/chemprop/chemprop (accessed on 7 October 2024))	Alpelisib (PI3K inhibitor) identified using ML tools for breast cancer	Requires extensive, high-quality datasets; risk of over-fitting.	[11,12]
Deep Learning (DL)	Screening drug candidates, predicting patient-specific drug responses, discovering hidden patterns	Handles large, complex datasets, enhances novel discovery	DeepChem 2.7.1, AtomNet (Atomwise, https://www.atomwise.com/ (accessed on 7 October 2024))	Lapatinib (EGFR/ErbB2 inhibitor) prediction improved with DL screening tools	DL models are “black boxes”, making them difficult to interpret; computationally expensive.	[13]
Natural Language Processing (NLP)	Mining literature and patents for drug discovery insights, identifying emerging drug trends	Extracts valuable information from unstructured data	SciBite (https://scibite.com/ (accessed on 7 October 2024)), TextMining (spaCy, https://spacy.io/ (accessed on 7 October 2024))	Identified Pembrolizumab in conjunction with other immune checkpoint inhibitors from literature mining	Struggles with domain-specific language; customization required to interpret scientific literature.	[14]
Generative Adversarial Networks (GANs)	Designing new chemical entities (NCEs), generating drug-like compounds with desired properties	Generates diverse and novel compounds, reduces reliance on traditional synthesis methods	MoleculeGAN (Academic Repos, https://github.com/ (accessed on 7 October 2024)), REINVENT (GitHub, https://github.com/MolecularAI/Reinvent (accessed on 7 October 2024))	Generated new EGFR inhibitors with properties for targeting cancer	Difficult to control quality of generated molecules; clinical validation often lacking.	[4]
Reinforcement Learning (RL)	Optimizing drug combinations, exploring synergistic effects, guiding decision-making in drug design	Provides adaptive learning, maximizes efficacy	Deep RL (Resources, https://github.com/ (accessed on 7 October 2024)), ChemTS (GitHub, https://github.com/tsudalab/ChemTS (accessed on 7 October 2024))	Combination of Vemurafenib and Cobimetinib for melanoma identified through RL	High computational requirements; accurate reward signals needed for clinical validation.	[15]
Quantum Computing (QC)	Simulating complex molecular interactions, optimizing quantum machine learning for faster discovery.	Solves complex computational chemistry problems.	Qiskit (https://www.ibm.com/quantum/qiskit (accessed on 7 October 2024)), IBM Quantum (IBM, https://quantum-computing.ibm.com/ (accessed on 7 October 2024))	Quantum simulations for Taxol drug interactions with tubulin in cancer treatment	Still in infancy with limited practical applications; scalability is a challenge.	[9]
AI for Biomarker Discovery	Identifying predictive biomarkers, facilitating personalized medicine, linking genetic profiles	Enhances personalized treatment strategies, improves patient selection for trials	OncoKB (https://oncokb.org (accessed on 7 October 2024)), BioX-press (https://bioxpress.org/ (accessed on 7 October 2024))	Identified biomarkers for Pembrolizumab effectiveness in melanoma	Large multi-omics datasets required; data privacy concerns and complex integration challenges.	[11,16]
AI-Based Virtual Screening	High-throughput screening (HTS) of drug libraries, accelerating lead identification	Increases speed and accuracy in identifying promising candidates	Schrödinger (https://www.schrodinger.com/ (accessed on 7 October 2024)), PyRx (https://pyrx.sourceforge.io/ (accessed on 7 October 2024)), VSpipe (GitHub, https://github.com/ (accessed on 7 October 2024))	Screened inhibitors for ERα (estrogen receptor) in breast cancer	Not always accurate predictor of in vitro success; requires follow-up validation.	[5,15]
AI in Drug Repurposing	Identifying existing drugs with potential anticancer properties, analyzing multidimensional data	Lowers costs, speeds up clinical trials, reduces risks	CANDO, DTC (Research Resources, https://github.com/ (accessed on 7 October 2024))	Repurposed Metformin as a potential anticancer agent for pancreatic cancer	Known toxicity profiles can limit repurposing; incomplete data can miss key interactions.	[12]
AI in Clinical Trial Optimization	Predicting patient outcomes, optimizing inclusion/exclusion criteria, improving trial design	Reduces trial costs, improves recruitment, enhances efficacy predictions	Deep 6 AI (https://www.deep6.ai (accessed on 7 October 2024)), IBM Watson for Clinical Trial Matching	Optimized trials for immunotherapy agents like Nivolumab	Ethical concerns in AI-driven patient selection; risk of introducing biases affecting trial diversity.	[17]

### 1.1. Background/History of AI Role in Drug Discovery and Development

#### 1.1.1. Evolution of AI: From Machine Learning (ML) to Deep Learning (DL)

AI has undergone a remarkable evolution over the last several decades, with its integration into drug discovery representing a significant shift in the way that pharmaceutical research is conducted. Initially, AI in drug discovery was driven by ML algorithms that could analyze structured data, recognize patterns, and predict outcomes based on statistical methods. Early AI techniques were limited by computational power and data availability, but they laid the groundwork for more sophisticated methods in later years [11,12,13,16,17,18].

The transition from ML to DL marked a significant leap in the field. While ML relies on algorithms designed to learn from data through human intervention, DL leverages artificial neural networks that are capable of learning directly from raw data with minimal human input. DL models, such as convolutional neural networks (CNNs) and recurrent neural networks (RNNs), have demonstrated a remarkable ability to process vast amounts of unstructured data, including images, genomics data, and chemical structures, making them particularly suited for tasks like drug discovery. The integration of DL into drug discovery processes has revolutionized the field, enabling predictive models with much higher accuracy, robustness, and adaptability compared to traditional methods. For instance, DL techniques have been instrumental in identifying novel drug targets, predicting drug–target interactions, and designing new compounds with optimized properties, accelerating the pace of drug development. Over time, advancements in computational power, coupled with the availability of large-scale biological datasets, have further fueled the expansion of AI in the pharmaceutical industry. As the scope of AI continues to grow, its integration into various stages of drug discovery—from initial target identification to preclinical and clinical testing—has paved the way for a new era of precision medicine, where treatments can be more personalized and targeted [13,17,18].

#### 1.1.2. AI’s Role in Accelerating Drug Development: From Early Target Discovery to Clinical Trials

The integration of AI technologies has become integral to each stage of the drug development pipeline. In the early stages, AI has proven invaluable in target identification and drug repurposing, where ML and DL algorithms are used to determine vast datasets for potential therapeutic targets and to predict how existing drugs could be repurposed for new indications. In the drug design phase, AI tools have significantly accelerated the process of hit identification, lead optimization, and de novo drug design. AI-driven algorithms, such as generative adversarial networks (GANs), are now used to create novel drug-like compounds by exploring chemical space far more efficiently than traditional methods. Additionally, AI models now play a crucial role in clinical trial design, where they assist in patient stratification, predicting patient responses, and identifying biomarkers that can indicate a drug’s efficacy or toxicity [14,15].

The ongoing integration of AI with reinforcement learning, natural language processing (NLP), and multi-omics data will enable even more precise drug discovery and personalized therapies. AI is expected to shorten the time to market for new drugs, reduce costs, and improve the overall efficiency of the drug development process [14]. In conclusion, the evolution of AI from early ML methods to advanced DL techniques has played an instrumental role in transforming drug discovery and development. As the technology continues to advance, it promises to further revolutionize the pharmaceutical industry, making drug development faster, more efficient, and more personalized than ever before.

## 2. AI Capability to Integrate Information from Diverse Sources

One of the key strengths of AI in the context of anticancer drug target identification is its ability to integrate data from multiple sources. Cancer is a complex disease with a multitude of underlying genetic, molecular, and cellular factors. Integrating data from various sources provides a more comprehensive understanding of these factors and helps in identifying potential drug targets [11]. AI algorithms can analyze genomic data to identify genetic mutations, alterations, and gene expression patterns associated with cancer. Integrating this data with other omics data (such as proteomics and transcriptomic) can reveal the molecular pathways driving cancer development. By combining data on protein expression and gene expression, AI can identify key proteins and their regulatory networks that are dysregulated in cancer cells. This helps in identifying targets that play a crucial role in disease progression. Integrating clinical data, including patient histories, treatment responses, and outcomes, with molecular data can provide insights into the effectiveness of different treatments for specific genetic profiles [12]. AI can integrate data from various sources to construct detailed signaling pathways and molecular networks. This enables researchers to pinpoint specific nodes within these networks that could be targeted with drugs. AI can analyze protein structures to predict how molecules interact with potential drug targets. Integrating structural data with other molecular data helps in understanding the mechanisms of DTIs [9,11]. AI can analyze data from high-throughput screening (HTS) assays to identify compounds that show activity against specific targets. Integrating screening data with other molecular information enhances target validation [16]. AI techniques like DL can fuse data from various sources to identify hidden patterns and relationships. This can reveal novel insights that may not be apparent when analyzing individual datasets [16].

An increasing number of approaches within similarity-based or data-driven frameworks aim to harness AI for enhanced predictive capabilities through the integration of diverse data types. Madhukar et al. [18] introduced a Bayesian-based ML method known as “BANDIT”, achieving approximately 90% accuracy in target prediction for over 2000 small molecules. The success was attributed to the integration of six data types, including growth inhibition, gene expression, adverse reactions, chemical structure, and drug-related data. In a similar vein, Olayan et al. [17], proposed the DDR method to explore more efficient predictions of DTIs through the utilization of data from multiple sources, encompassing eight drug similarity networks and eight target similarity networks. The drug similarity networks comprised various elements such as gene expression similarity, disease-based similarity, drug side effect–based similarity, and chemical structure fingerprint-based similarity. Similarly, the target similarity networks encompassed features like gene ontology–based similarity and protein sequence–based similarity. These investigations highlight that leveraging AI to integrate data from diverse sources enhances both the biological interpretability of drug target prediction and prediction accuracy [13].

## 3. AI in Anticancer Drug Target Identification

AI has made significant contributions to various aspects of cancer research, including drug target identification [6,9]. Identifying suitable drug targets is a critical step in the drug discovery process, as it helps researchers to design therapies that can specifically target cancer cells while minimizing detriment to healthy cells. AI techniques have been particularly valuable in this area due to their ability to analyze large and complex biological datasets and identify patterns that might be difficult for humans to discern [15]. Drug–target interaction (DTI) is a key step in drug development. The robustness of the interaction between a drug and its target is frequently characterized by binding affinity constants, which encompass metrics such as the dissociation constant (Kd), inhibition constant (Ki), and half-maximal inhibitory concentration (IC_50_) [14]. The experimentally determining DTI is a resource-intensive and time-consuming endeavor, and the computational prediction of these interactions holds considerable significance. Precise and efficient DTI predictions have the potential to significantly enhance drug development processes and expedite the discovery of lead or hit compounds [19].

Computational approaches for predicting DTIs encompass methods such as molecular docking simulations and those grounded in ML. An innovative end-to-end learning framework named “EEG-DTI” for DTI predictions was introduced by Peng et al., leveraging heterogeneous graph convolutional networks [20]. This model employed a graph convolutional network to acquire low-dimensional feature representations of drugs and targets, facilitating DTI prediction based on the learned features. Notably, it demonstrated strong DTI prediction performance, even in cases where the three-dimensional structures of drug targets were not employed. For enhanced prediction accuracy, Shao et al. treated DTI prediction as a link prediction challenge and introduced an end-to-end model called “DTI-HETA”, utilizing a heterogeneous graph with an attention mechanism [21]. This model surpassed the performance of current state-of-the-art models. Simultaneously, in tackling the interpretability challenge of DL, Yang et al. presented a method for predicting DTIs. This method relied on mutual learning mechanisms, operated without 3D structural data, and provided explanatory insights [22].

## 4. AI Predicts the Viability of Drug Targets for Anticancer Therapies

The selection of drug targets is also a very crucial step in the cancer drug design process. AI can play a significant role in predicting the druggability of potential anticancer drug targets. Druggability refers to the likelihood that a target can be modulated by a small drug molecule in a way that produces a therapeutic effect. Identifying druggable targets is important in the drug discovery process, as it helps researchers to prioritize which targets are more likely to lead to successful drug development [23]. AI-driven virtual screening involves the computational screening of large databases of chemical compounds to identify those that are likely to bind to a target of interest. This approach can help researchers identify potential drug candidates that could modulate anticancer targets. AI can predict the 3D structure of proteins and analyze their binding sites. This enables researchers to assess whether a target’s structure is suitable for small molecule binding. Structural information helps in understanding how a potential drug molecule could interact with the target. AI models can predict the binding affinity between a target protein and potential drug molecules. This helps in determining whether a small molecule is likely to bind strongly to the target, a key factor in drug development [24]. Numerous AI related methodologies have been devised (Table 2). Raies et al. [25], introduced a predictive model named “DrugnomeAI” to tackle the challenge of targeted drug synthesis. They employed a stochastic semi-supervised ML framework to develop DrugnomeAI for forecasting the druggability of drug targets within the human exome. Additionally, the study showcased the capability of DrugnomeAI in predicting the druggability of drug targets specifically in oncology diseases. In a separate effort, Wang et al. [26] formulated a novel model, KG4SL, grounded in a graph neural network (GNN). This model integrates knowledge graph (KG) messaging into GNN predictions. The empirical findings underscored the substantial positive impact of incorporating KG into GNN for SL prediction.

## 5. AI Screening of Potential Hit Compounds for Anticancer Drugs

AI holds a crucial position in assessing potential compounds for anticancer drugs. Within the field of drug discovery, the objective is to identify molecules that can interact with a target protein, positively influencing its function for disease treatment [35]. AI acts as an essential tool in this process, aiding in the prediction and optimization of compounds with increased affinity and specificity for cancer-related targets. As a result, AI significantly accelerates and improves the drug discovery process [36]. Integrating AI into hit compound screening not only speeds up the discovery of potential drug candidates but also reduces the costs and time associated with experimental screening. Once therapeutic targets for anticancer drugs are identified, the subsequent phase involves screening for hit compounds—molecules exhibiting initial activity against a specific target or pathway [37]. The computer-aided exploration of hit compounds is primarily facilitated through HTS, which can be carried out using two main approaches: structure-based screening and ligand-based screening [27].

### 5.1. Strategies for Structure-Based Screening

The structure-based approach relies on known structural information to characterize the interaction effects between bioactive compounds and their corresponding receptors [38]. Advancements in bimolecular spectroscopic technologies, such as X-ray crystallography and nuclear magnetic resonance (NMR), have significantly enhanced our understanding of the drug target’s structure. Leveraging the three-dimensional structure of proteins, structure-based design (SBD) allows for the rational design of new ligands to elicit therapeutic effects. SBD offers crucial insights into the discovery and optimization of initial lead compounds for new drug design and development [39,40]. High-affinity ligands, guided by SBD, selectively regulate validated drug targets, influencing specific cellular activities and ultimately achieving the desired pharmacological and therapeutic effects [41].

In the realm of anticancer drug design, structure-based virtual screening utilizes docking and scoring techniques to identify molecules exhibiting robust binding affinity for a target protein [42]. Nevertheless, conventional docking procedures are frequently time-consuming, presenting challenges for extensive virtual screening. To address this issue, Lu et al. integrated DL models into structure screening, developing a model capable of predicting molecular docking scores and accelerating the evaluation process [43]. Yasuo et al. introduced an innovative structure-based virtual screening approach for hit compounds, known as “SIEVE-Score”, which harnesses AI and showcases substantial improvements compared to other contemporary virtual screening methods [44].

#### 5.1.1. Molecular Docking

Molecular docking, although not inherently rooted in AI, is a computational method extensively applied in drug discovery, notably in the context of anticancer drug research. This technique involves predicting the preferred orientation and conformation of a ligand (a drug molecule) when it binds to a target protein. The assessment encompasses the strength and geometry of the interaction between the ligand and the target, providing valuable insights into potential binding sites and affinities [45]. While traditional molecular docking relies on physics-based scoring functions and algorithms for predicting binding interactions, recent strides in drug discovery involve the integration of AI techniques to augment the precision and efficiency of docking studies. Approaches grounded in AI, such as ML and DL, have the potential to enhance scoring functions, refine predictions, and address intricate interactions in the context of molecular docking [46]. By leveraging extensive datasets, these techniques can train models that more effectively capture the subtleties of molecular interactions. In essence, while molecular docking stands as a prevalent computational technique in anticancer drug discovery, the integration of AI methods into this domain holds promise for elevating the predictive capabilities and precision of docking studies. Integrating AI techniques into molecular docking for computational drug discovery entails utilizing ML and other AI methods to improve the precision and efficiency of docking studies [47].

#### 5.1.2. Integrating Molecular Docking with AI for Comprehensive Processing

ML models have significantly improved scoring mechanisms in molecular docking, enhancing the accuracy of drug discovery processes. These models are trained on diverse datasets to comprehend complex relationships between molecular features and binding affinities, thereby providing more reliable scoring functions. In particular, regression-based ML models are highly effective in predicting binding affinities with greater precision. By utilizing experimental binding affinity data, these models can recognize intricate patterns and correlations, leading to more accurate outcomes [48]. DL techniques further contribute by automatically extracting relevant features from molecular structures. Neural networks develop hierarchical representations, capturing fine details crucial for docking accuracy. As a result, DL has become instrumental in improving docking performance by providing a more nuanced understanding of molecular interactions [49]. In addition to enhancing scoring functions, ML algorithms optimize virtual screening processes. By prioritizing compound libraries based on predicted binding affinities, these algorithms significantly improve the efficiency of hit identification. Another key advancement is in handling protein flexibility during docking, as AI-driven methods can accommodate conformational changes in target proteins [50]. This is often achieved through molecular dynamics simulations or ML models capable of predicting protein flexibility. Generative models based on DL are also making strides in ligand design. These models can craft novel ligands with specific properties, suggesting new chemical structures for synthesis and testing. Transfer learning techniques further streamline this process by leveraging knowledge from existing docking studies, allowing pretrained models to be fine-tuned for specific targets, thereby reducing the need for extensive training data [51]. Ensemble models, which combine predictions from multiple docking runs or AI models, provide a robust and high-performing approach. The integration of AI predictions with experimental data helps refine and validate docking results, ensuring that predictive power is continuously improved [52]. Real-time adaptation is another promising development, where adaptive algorithms adjust docking parameters based on ongoing experimental feedback, allowing for dynamic optimization [9,16]. Overall, incorporating AI techniques into molecular docking workflows enhances the accuracy, speed, and reliability of computational drug discovery efforts. This integration ultimately facilitates the identification and optimization of potential drug candidates, offering a powerful tool for modern drug development [53].

#### 5.1.3. Structure-Based Pharmacophore Mapping

Pharmacophore mapping is the process of discerning the critical features of a molecule that contribute to its biological activity, enhancing our comprehension of the interactions between a drug and its target. Although not originally an AI technique, the application of AI-based methods in pharmacophore mapping accelerates drug discovery by efficiently analyzing extensive datasets and predicting potential pharmacophores more swiftly [54]. In the realm of anticancer drug discovery, AI-based methods become pivotal in pharmacophore mapping, utilizing ML and data analysis.

#### 5.1.4. Integration of AI in Pharmacophore Mapping

AI has transformed the drug discovery process by enabling the analysis and extraction of critical data features from extensive molecular datasets. Through the use of AI algorithms, researchers can analyze vast datasets containing molecular structures and biological activities to identify key features associated with anticancer properties. ML models are particularly effective in extracting crucial molecular descriptors or fingerprints that contribute to the pharmacophore of effective anticancer drugs. This process allows for a more targeted and efficient approach to drug discovery [55]. One of the most significant applications of AI in this field is virtual screening, where AI predicts the binding affinity of compounds to cancer-related targets [14,15]. DL models, leveraging insights from known molecular interactions, can identify potential ligands that meet pharmacophoric requirements necessary for anticancer activity [24]. This screening process enhances the chances of discovering potent drug candidates. Generative models, such as generative adversarial networks (GANs) and variational autoencoders (VAEs), further boost drug discovery efforts by generating novel molecular structures with desired pharmacophoric features. These AI-driven models create new possibilities for drug design, generating compounds that are tailored to meet specific therapeutic needs [56]. AI also excels in integrating multi-omics data, including genomics, proteomics, and metabolomics, providing a comprehensive understanding of cancer at the molecular level. This integration aids researchers in identifying relevant pharmacophores, thus contributing to more accurate and effective anticancer drug design [57]. Moreover, AI models are capable of predicting the absorption, distribution, metabolism, excretion, and toxicity (ADMET) properties of potential compounds. This capability ensures that drug candidates not only demonstrate anticancer activity but also possess favorable pharmacokinetic profiles for further development [27,33]. In addition to these advancements, AI accelerates drug design through reinforcement learning and optimization algorithms, enabling the creation of new compounds with optimized pharmacophoric features. This significantly speeds up the drug discovery process by efficiently navigating large chemical spaces to identify promising candidates. By combining AI methodologies with pharmacophore mapping, researchers can streamline the development of anticancer drugs, making the entire process faster and more precise [58].

### 5.2. Ligand-Based Pharmacophore Mapping in Drug Discovery

Ligand-based screening involves the identification of small molecules with known activities and the exploration of structures within a compound library exhibiting similar physical or chemical characteristics as potential candidates. The fundamental principle underlying ligand-based approaches in drug discovery is molecular similarity. These methods rely on the structural information of active ligands interacting with the target protein, using a compound with noteworthy biological properties as a template to identify and predict new chemical entities with comparable properties [59]. This methodology is considered an indirect protocol for drug discovery, relying solely on the structure of known active small molecules, without the need to predict the 3D protein structure. The approach is commonly employed to virtually screen novel ligands with intriguing biological activities and to optimize the biological properties of ligands, enhancing drug pharmacokinetics, including ADMET. Ligand based pharmacophore mapping is a crucial aspect of drug discovery that involves the identification and characterization of molecular features essential for a ligand to interact with a target protein [60]. Ligand-based pharmacophore mapping focuses on the properties of known active ligands and their spatial arrangement, aiming to understand the key elements responsible for biological activity.

#### 5.2.1. AI Integrated Software Tools for Ligand-Based Pharmacophore Mapping

Various computational tools are available for pharmacophore mapping, such as Ligand Scout, MOE (Molecular Operating Environment), and Discovery Studio. These tools assist in the generation, visualization, and validation of pharmacophores [27,54]. Ligand-based pharmacophore mapping, when integrated with other computational and experimental techniques, significantly contributes to the rational design of new drugs with improved potency and selectivity. It plays a vital role in streamlining the drug discovery process and reducing the reliance on serendipity in identifying potential therapeutic agents [61]. Krasoulis et al. introduced an end-to-end approach called “DENVIS”, which presents a scalable and innovative algorithm for HTS. This approach utilizes graphical neural networks incorporating atomic and surface protein pocket features. In experiments on two benchmark databases, DENVIS demonstrated significantly improved speed compared to other models [62]. Turkina et al. recently developed a cumulative molecular fingerprinting algorithm that comprehensively considers all structural data in the calculation, effectively enhancing the utilization of experimental data. This method achieves a seamless integration of molecular fingerprinting and experimental information, inheriting the speed advantage of the former approach while achieving higher information utilization [63].

#### 5.2.2. Ligand-Based Quantitative Structure–Activity Relationship (QSAR) Modeling

QSAR modeling is a computational approach used in drug discovery to establish relationships between the chemical structure of molecules (ligands) and their biological activities [47]. Ligand-based QSAR specifically focuses on the properties and features of ligands with known bioactivity against a target [55]. The traditional QSAR approach involves statistical methods to correlate physicochemical properties or molecular descriptors with biological activities [64]. These descriptors capture information about the structural features of molecules, allowing researchers to predict how changes in chemical structure might affect a compound’s activity. In recent years, AI, particularly ML, has been integrated into QSAR modeling to improve accuracy and efficiency. ML algorithms can handle large datasets, identify complex patterns, and make predictions without explicitly programmed rules [65]. This synergy has led to the development of QSAR models with enhanced predictive power. In summary, while QSAR modeling itself is not purely an AI tool, the integration of AI techniques has significantly advanced its capabilities in drug discovery by enabling more accurate predictions and handling larger and more complex datasets [66].

#### 5.2.3. Integration of AI Tools with QSAR Modeling

QSAR modeling involves establishing mathematical relationships between the chemical structure of compounds and their biological activities. The integration of AI tools with QSAR modeling enhances the efficiency, accuracy, and predictive power of these models. The integration of AI tools with QSAR modeling has significantly transformed traditional methods, making the modeling process more efficient, accurate, and scalable. In conventional QSAR modeling, data preprocessing involves manual calculation of descriptors, handling missing values, and dealing with data outliers [67]. With AI integration, these steps can be automated and optimized, allowing AI tools to handle large datasets efficiently, including imputing missing values and selecting relevant descriptors with minimal human intervention. When it comes to descriptor selection, the traditional approach relies on domain knowledge or statistical methods to manually choose relevant descriptors. In contrast, AI, particularly ML, can automatically identify and prioritize descriptors by recognizing complex relationships in the data. Techniques like recursive feature elimination become more powerful through AI, ensuring more precise and relevant feature selection. Model building in traditional QSAR is often based on linear regression or similar methods, which may struggle to capture nonlinear relationships in complex biological systems [68]. AI integration, through ML and DL, offers greater flexibility in modeling nonlinear relationships between molecular descriptors and biological activities. Neural networks, a DL technique, are particularly useful in uncovering intricate patterns within data that are not apparent through conventional methods such as partial least squares or support vector machines. AI tools significantly improve prediction accuracy by learning complex dependencies and patterns in data, which traditional QSAR methods, constrained by linearity assumptions, often miss. Transfer learning further enhances model performance by allowing models trained on one dataset to be fine-tuned for use on another, thereby leveraging knowledge across datasets and improving generalization [69]. In addition to improving prediction accuracy, AI automates the model selection process. While traditional approaches require manual selection based on statistical metrics, AI-driven algorithms can explore a wider range of models, optimizing for both accuracy and interpretability. This is especially valuable when dealing with large datasets, where AI’s scalability, including the use of distributed computing and cloud-based solutions, allows for efficient handling of big data challenges.

AI’s potential to address big data challenges in QSAR modeling extends far beyond traditional methods. Unlike conventional QSAR, where data handling and modeling are often limited by linear approaches, AI-based techniques like ML and DL can process high-dimensional data with intricate patterns [47]. These methods enable faster processing and feature selection by utilizing algorithms capable of identifying meaningful descriptors from complex datasets, even with significant noise or missing values. Furthermore, AI facilitates distributed computing, allowing models to scale across vast molecular databases and provide real-time adaptability as new data becomes available [55]. This adaptability and scalability make AI an indispensable tool for handling the high volume, variety, and velocity of big data in QSAR, leading to faster, more precise predictive models and more effective drug discovery efforts. AI facilitates distributed computing, which allows models to scale across vast molecular databases and handle large volumes of data that are beyond the capacity of conventional computing systems. This distributed approach enhances the ability to process data in parallel, speeding up the modeling process and improving real-time adaptability as new data becomes available. As a result, AI models can continuously learn and evolve, adjusting their predictions based on fresh inputs and improving over time. The scalability and adaptability of AI are essential for handling the high volume, variety, and velocity of big data in QSAR modeling. In drug discovery, where datasets often include millions of compounds and experimental results, AI’s capacity to process and analyze such data efficiently leads to faster development of more accurate predictive models. This, in turn, accelerates the identification of promising drug candidates, ultimately driving more effective drug discovery and development processes. AI’s ability to tackle big data challenges is transforming QSAR modeling, making it a more powerful tool for advancing medicinal chemistry and material science [70].

Moreover, AI allows for continuous learning, where models adapt and evolve as new data becomes available, ensuring that they remain relevant over time. Overall, the integration of AI tools with QSAR modeling enhances the entire workflow, from data preprocessing to model building and prediction accuracy. It enables more robust and adaptable predictive models, offering new possibilities for understanding complex structure–activity relationships in drug discovery and material science [71]. The synergy between AI and QSAR modeling is transforming how researchers develop more accurate and efficient models in these fields.

## 6. Molecular Dynamics (MD) Simulation in Finding New Drug Binding Sites

MD simulations play a pivotal role in drug discovery by providing a dynamic and detailed view of molecular interactions within biological systems. Understanding the dynamics of proteins and their interactions with ligands is crucial for identifying new drug binding sites [13]. MD simulations play a crucial role in drug discovery by capturing the dynamic behavior of biomolecular systems, providing insights beyond static structures. They enable the exploration of protein structures over time, revealing flexibility and conformational changes that can uncover hidden or transient binding sites [42,48]. MD simulations are instrumental in characterizing ligand binding pathways, identifying how ligands approach and bind to target proteins, and detecting allosteric binding sites—regions that modulate protein function despite not being part of the active site [72]. They also capture transient binding events and predict cryptic binding sites, which are inaccessible in experimental structures but may become available under specific conditions like changes in pH or temperature. MD simulations are invaluable for validating and refining computational models, such as docking studies, by confirming binding site predictions and assessing the stability of ligand–protein interactions [73]. Furthermore, MD provides insights into binding site flexibility, showing how sites adapt to different ligands, and can quantify binding affinities and kinetics by analyzing interaction energetics. By integrating MD results with experimental techniques like X-ray crystallography and NMR spectroscopy, researchers can refine models and validate binding sites, providing a comprehensive and dynamic perspective that enhances rational drug design. In sum, MD simulations significantly improve the discovery of drug binding sites by offering a detailed, time-dependent view of biomolecular interactions, making them a powerful tool in modern drug discovery [74].

### Integration of MD Simulation with AI

The integration of AI with MD simulations revolutionizes the process of identifying new drug binding sites by combining the strengths of both technologies. MD simulations, which involve solving Newton’s equations of motion to simulate molecular movements over time, provide detailed insights into the dynamic behavior and interactions of biomolecules. When integrated with AI-driven techniques like ML and DL, the efficiency of these simulations is enhanced. AI algorithms can extract relevant features from MD data, build predictive models, and identify conformational changes that reveal hidden binding sites [75]. Hybrid approaches combining physics-based MD simulations with data-driven AI methods offer a comprehensive strategy for understanding complex biological processes [76]. Tools like Markov state models (MSMs) benefit from AI by improving the identification of key conformational changes, while clustering techniques and principal component analysis (PCA) are enhanced through AI to better analyze MD data and reveal stable binding site conformations. AI also strengthens the predictive power of drug binding site prediction tools like Sitemap and FT-Map, and convolutional neural networks (CNNs) are used to recognize complex patterns in MD trajectory frames, aiding in binding site discovery [77]. Furthermore, integrating MD with ensemble docking, where multiple protein conformations are used for ligand docking, and leveraging quantum mechanics/molecular mechanics (QM/MM) simulations allow for more accurate and insightful representations of ligand–target interactions. Overall, AI-driven MD simulations offer increased efficiency, accuracy, and the potential to uncover novel drug binding sites [25,72]. However, challenges such as the computational demands of these simulations and the reliance on high-quality data for ML models need to be addressed to fully harness their potential in drug discovery [78].

## 7. Identification of Anticancer Drugs Using AI-Based De Novo Drug Design

AI-based de novo drug design is a powerful approach for identifying anticancer drugs by creating new molecules with desired properties through computational methods, without relying on existing compounds. This method leverages various computational techniques to design novel compounds with therapeutic potential [30]. Molecular docking and virtual screening are commonly employed to predict how small molecules might bind to cancer-related proteins, helping to identify promising candidates. QSAR modeling aids in correlating molecular structures with anticancer activity, while fragment-based drug design combines smaller fragments to form effective drug molecules. Pharmacophore modeling and ligand-based virtual screening focus on identifying molecular frameworks and similar structures from known anticancer compounds to discover new drug candidates [79]. Structure-based design methods analyze the three-dimensional structures of proteins to design molecules that fit well in binding sites. AI-driven ML techniques analyze large chemical and biological datasets, predicting the anticancer potential of new molecules by identifying complex patterns. Additionally, fragment-based lead discovery screens libraries of fragments to create new lead compounds, while genetic algorithm-based design simulates evolutionary processes to optimize molecules for anticancer activity [80]. Multiobjective optimization balances factors like potency and selectivity to develop molecules with an overall favorable profile. Advanced computational power, combined with HTS and virtual screening (VS), allows for the efficient evaluation of vast compound libraries. AI techniques such as variational autoencoders (VAEs), recurrent neural networks (RNN), generative adversarial networks (GAN), and deep reinforcement learning (DRL) are employed to generate and optimize new molecules, making de novo drug design a crucial tool for modern anticancer drug discovery [81].

## 8. Role of AI in Anticancer Drug Repurposing

### 8.1. Overview of Anticancer Drug Repurposing

Drug repurposing, often referred to as drug repositioning, identifies alternative therapeutic uses for medications that have already been approved for other conditions, offering the potential to treat complex diseases like cancer. This approach allows researchers to circumvent the extensive time and financial resources required for new drug development by leveraging existing knowledge about these drugs’ safety profiles, pharmacokinetics, and mechanisms of action. Many medications originally designed for specific ailments exhibit additional therapeutic effects because they interact with diverse biological pathways that impact multiple diseases. Consequently, drug repurposing has attracted growing attention in recent years for its ability to accelerate the drug discovery process and offer new treatments for cancer [31,82]. Despite extensive research efforts in academia and the pharmaceutical industry, current anticancer therapies have shown substantial success in only a limited number of cancer types, underlining the need for innovative approaches like drug repositioning. Drug repurposing strategies fall into two primary categories: target-centric and disease-centric approaches, aimed at predicting novel drug–target and drug–disease interactions, respectively. These strategies not only streamline the drug discovery process but also broaden the range of potential therapeutic options for patients, supporting a more efficient and resource-saving paradigm in cancer treatment [83,84].

### 8.2. AI-Driven Insights into Drug–Target Interactions

**Figure 2:**
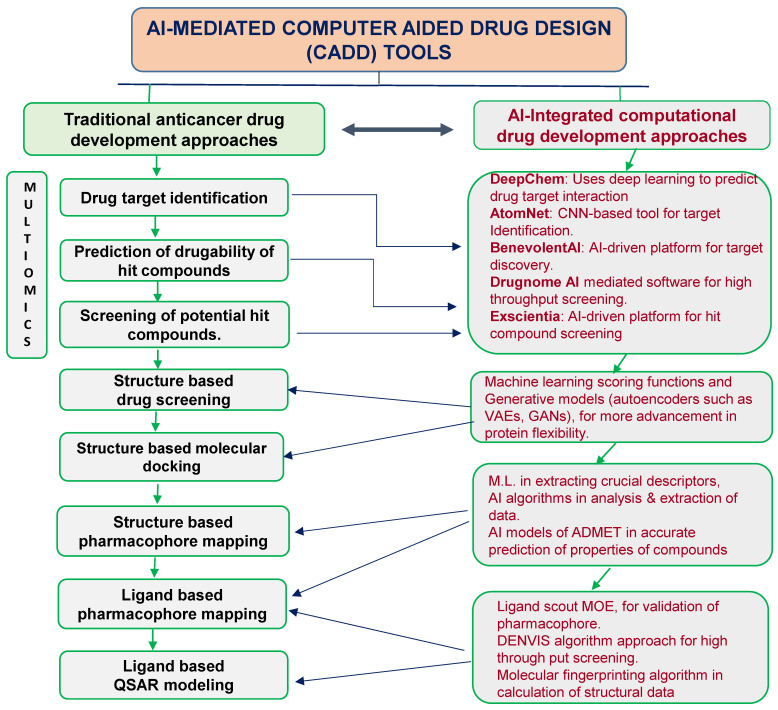
AI-mediated computer-aided drug design (CADD) tools: Integration of traditional and AI-driven approaches in anticancer drug development. The figure showcases how AI revolutionizes traditional drug discovery by leveraging advanced computational power and data-driven insights. On one side, the traditional multi-omics process is depicted, highlighting key steps such as drug target identification, druggability prediction, hit compound screening, molecular docking, pharmacophore mapping, and QSAR modeling. On the other side, AI-driven tools like DeepChem, AtomNet, BenevolentAI, Drugnome AI, and Exscientia enhance speed and precision in drug design. AI models improve scoring functions, protein flexibility, and ADMET predictions, while tools like LigandScout MOE and DENVIS Algorithm add value to pharmacophore validation and structural data analysis. Together, these innovations bridge the gap between computational predictions and experimental validation, accelerating drug discovery.

AI has become an essential tool in drug repositioning, particularly by elucidating complex interactions between drugs and new biological targets. By analyzing massive datasets generated from biological studies, clinical trials, and chemical properties, AI methodologies like ML and DL have transformed our ability to discover alternative therapeutic applications for approved medications [85]. For example, predictive modeling can assess interactions between drugs and targets by examining molecular structures and known drug–target relationships. Through computational techniques such as molecular docking and virtual screening, AI can predict a drug’s binding efficacy with potential new targets. This enables a faster identification of drug candidates that might be effective against complex diseases like cancer, where traditional development methods often fall short [86]. Moreover, AI facilitates the integration of multi-omics data—encompassing genomics, proteomics, metabolomics, and transcriptomics—giving researchers a comprehensive understanding of disease processes and associated biological pathways. This data-driven, holistic approach is crucial for complex diseases like cancer, where numerous interconnected pathways contribute to disease progression, thereby enabling AI to identify both direct drug–target interactions and indirect effects, which can lead to novel therapeutic advantages [28] (Figure 2).

### 8.3. Enhancing Data Analysis and Clinical Trials Through AI

Another significant advantage of AI in drug repurposing is its capacity to improve the speed, accuracy, and depth of data analysis. For instance, natural language processing (NLP) algorithms can sift through vast amounts of scientific literature and clinical trial data, extracting insights into drug interactions and potential new uses that may have previously been overlooked [87]. By continuously learning and updating with new data, AI systems adapt their predictions, thereby increasing the reliability of drug repurposing efforts. Beyond analysis, AI contributes to experimental design in preclinical and clinical trials by predicting which drug candidates are most likely to succeed based on historical and real-time data. By doing so, AI can streamline the clinical trial process, reducing time and associated costs, and ultimately accelerating the path to market for new therapies. This predictive capability not only improves the efficiency of drug development but also enhances the chances of clinical success by optimizing the selection and prioritization of repurposed drugs for further investigation [32]. In summary, the integration of AI into drug repurposing represents a groundbreaking shift in pharmaceutical research, as it augments our understanding of drug mechanisms, expedites discovery processes, and offers promising new avenues for cancer treatment [29,34,88,89,90].

## 9. Conclusions and Future Perspectives

In summary, the integration of AI into the realm of computational anticancer drug discovery is not merely an enhancement; it is a paradigm shift that promises to redefine the landscape of cancer therapeutics. Through advanced algorithms and data-driven methodologies, AI systematically addresses the myriad challenges inherent in the drug discovery pipeline, from the initial stages of target identification to the intricate processes of clinical trial optimization [34]. AI accelerates the identification of novel drug candidates, deepens our understanding of complex biological interactions, and facilitates the development of personalized treatment strategies tailored to individual patient profiles. By employing sophisticated ML models, AI predicts drug–target interactions with remarkable accuracy, designs optimized drug structures, and identifies opportunities for repurposing existing therapies for anticancer applications [89]. Moreover, AI-driven patient stratification allows for the implementation of tailored therapies, thereby maximizing treatment efficacy and improving patient outcomes. Its ability to analyze and extract valuable insights from vast omics datasets enhances the identification of biomarkers and elucidates mechanisms of drug resistance. Additionally, AI streamlines literature mining through natural language processing, ensuring that researchers remain at the forefront of the latest advancements in cancer research. Automation of high-throughput screening further accelerates data analysis, facilitating the rapid identification of promising drug candidates. However, while AI is an extraordinarily powerful tool, it is imperative to rigorously validate its predictions through experimental and clinical studies to ensure their reliability and applicability [29]. Ethical considerations, transparency, and the mitigation of biases within AI algorithms are critical areas that require ongoing scrutiny and refinement. Ultimately, the fusion of AI with computational anticancer drug discovery significantly enhances the pace of innovation, boosts research efficiency, and ushers in a more targeted and personalized approach to cancer treatment. As this dynamic field continues to evolve, fostering collaboration between AI technologies and traditional research methodologies will be essential to translating computational insights into clinically impactful and safe anticancer therapeutics [90]. Looking ahead, the synergistic potential of AI and cancer research could hold the promise of unlocking new avenues for effective cancer therapies, paving the way for breakthroughs that could transform patient care and outcomes.

## 10. Clinical Impact

- AI-driven models enhance the ability to predict patient-specific drug responses, enabling personalized treatment plans. This results in more precise and effective cancer therapies, reducing adverse effects and improving patient outcomes.
- AI expedites the screening and optimization of anticancer compounds, significantly shortening drug development timelines. This leads to faster clinical implementation of novel therapies, offering new treatment options for cancer patients.

## 11. Significance

AI-driven computational approaches for transforming anticancer drug development and prediction hold transformative potential by streamlining the drug discovery process with unprecedented speed and accuracy. AI enables rapid identification of novel compounds and optimizes treatment efficacy, significantly reducing development costs and failure rates. Moreover, AI’s ability to predict patient-specific responses paves the way for personalized cancer therapies, offering more targeted and effective treatment options, ultimately improving clinical outcomes for cancer patients.

## Figures and Tables

**Table 2:** Overview of AI-integrated software tools and techniques in anticancer drug design: Applications, advantages, challenges, and case-study references.

AI Application in Anticancer Drug Design	Description	Key Advantages	Challenges and Limitations	AI-Integrated Software Tools (Version/Access Details) *	References
Drug–Target Interaction Prediction	AI algorithms predict potential interactions between drugs and their targets	Increases the speed of identifying viable drug candidates; enhances accuracy of predictions	Requires large, high-quality datasets; predictions can be biased based on training data; overfitting is a risk if not managed properly.	Chemoinformatics Software: BindingDB (Version 2023.09, accessed November 2024) Docking Tools: AutoDock (Version 4.2.6, accessed November 2024); MOE (Version 2023.09, subscription)	[22]
Compound Screening and Optimization	AI methods screen vast chemical libraries to identify promising candidates	Reduces time and costs associated with traditional high-throughput screening methods	Virtual predictions may not always correlate with in vitro results; requires thorough experimental validation.	Virtual Screening Platforms: Schrödinger Suite (2024.2, accessed November 2024); DeepChem (Version 2.7.1, accessed November 2024)	[27]
Patient-Specific Drug Response Prediction	AI models analyze patient data to predict individual responses to specific drugs	Facilitates personalized medicine; helps in tailoring treatments for better outcomes	Data privacy concerns; requires comprehensive patient data and validation; risk of misclassification based on model bias.	Predictive Modeling Tools: IBM Watson for Drug Discovery (Updated 2023, subscription-based); Tempus (Platform details, https://www.tempus.com accessed November 2024)	[28]
Biomarker Discovery	AI identifies potential biomarkers that predict responses to therapies	Enhances patient stratification; supports the development of personalized treatment plans	Requires integration of multi-omics data; potential ethical concerns regarding genetic data usage.	Bioinformatics Software: GenePattern (Version 3.9.0, accessed November 2024); CBioPortal (Version 2024.10, open access)	[29]
De Novo Drug Design	AI generates novel chemical entities that can act as new anticancer drugs	Accelerates the discovery of innovative compounds; opens up new avenues for drug discovery	Generated compounds may lack drug-like properties; quality control of generated structures is crucial.	Generative Design Tools: DeepGen (Platform, https://github.com/ details not disclosed); MolecularAI (Proprietary, inquire at MolecularAI site, https://www.molecularai.com/)	[30]
Drug Repurposing	AI analyzes existing drugs for new anticancer applications	Reduces development costs and timelines; known safety profiles can expedite clinical trials	Limited by existing drugs’ toxicity profiles; AI may overlook some interactions due to data limitations.	Repurposing Platforms: Drug Repurposing Hub (Open Access, curated, accessed November 2024); RepoDB (Free, [Version 2024])	[31]
Clinical Trial Design Optimization	AI optimizes trial protocols, including patient selection and endpoint definitions	Improves recruitment efficiency; enhances trial success rates and reduces timelines	Ethical concerns related to AI-driven patient selection; requires careful validation against traditional trial designs.	Trial Optimization Tools: Trialspark (Platform Details, version not specified); Medidata (Version 2024.10, subscription required)	[32]
Toxicity Prediction	AI models assess the potential toxicity of new compounds early in the design process	Reduces the likelihood of late-stage failures in clinical trials due to safety issues	High false-positive rates can occur; requires extensive toxicology datasets for accurate predictions.	Toxicity Prediction Software: DEREK Nexus (Version 7.0, proprietary); DeepTox (Accessed via GitHub, https://github.com/DeepTox)	[33]
Integration of Multi-Omics Data	AI integrates genomic, proteomic, and metabolomic data to provide comprehensive insights	Facilitates understanding of complex cancer biology; enhances target identification	Data integration challenges; requires advanced computational resources; may face data heterogeneity issues.	Multi-Omics Platforms: GATK (Version 4.4.0, open source); OmicsHub (Commercial access)	[12]
Real-Time Monitoring of Trials	AI technologies enable continuous monitoring of trial data and patient responses	Facilitates adaptive trial designs; allows for real-time adjustments based on findings	Relies on the availability of real-time data; requires robust data infrastructure and ethical considerations for patient privacy.	Real-Time Monitoring Tools: Medidata Rave (Commercial Suite, updated November 2024); Clinical Ink (Contact for Info, https://clinicalink.com)	[34]

* Accessed Dates: November 2024 for all open-access tools and platforms.

## Data Availability

No new data was created or analyzed in this study. Data sharing is not applicable to this article.

## Abbreviations

ADMET, Absorption, Distribution, Metabolism, Excretion, Toxicity; AI, Artificial Intelligence; ANN, Artificial Neural Network; CADD, Computer-Aided Drug Design; CNN, Convolutional Neural Network; CTD, Clinical Trial Design; DL, Deep Learning; DNN, Deep Neural Network; DTI, Drug–Target Interaction; GAN, Generative Adversarial Network; GPCR, G-Protein-Coupled Receptor; HTS, High-Throughput Screening; LSTM, Long Short-Term Memory (neural networks); MD, Molecular Dynamics; ML, Machine Learning; NCE, New Chemical Entity; NLP, Natural Language Processing; NN, Neural Network; QSAR, Quantitative Structure–Activity Relationship; QC, Quantum Computing; RNN, Recurrent Neural Network; RL, Reinforcement Learning; SBD, Structure-Based Design; SVM, Support Vector Machine; TCR, T-Cell Receptor; VAE, Variational Autoencoder; VS, Virtual Screening.
